# The citrus flavanone naringenin impairs dengue virus replication in human cells

**DOI:** 10.1038/srep41864

**Published:** 2017-02-03

**Authors:** Sandra Frabasile, Andrea Cristine Koishi, Diogo Kuczera, Guilherme Ferreira Silveira, Waldiceu Aparecido Verri, Claudia Nunes Duarte dos Santos, Juliano Bordignon

**Affiliations:** 1 Sección Virologia, Facultad de Ciencias, Universidad de La República, 11400, Montevideo, Uruguay; 2 Laboratório de Virologia Molecular, Instituto Carlos Chagas, ICC/FIOCRUZ/PR, Curitiba, Paraná, Brazil; 3 Departamento de Ciências Patológicas, Centro de Ciências Biológicas, Universidade Estadual de Londrina, Paraná, Brazil

## Abstract

Dengue is one of the most significant health problems in tropical and sub-tropical regions throughout the world. Nearly 390 million cases are reported each year. Although a vaccine was recently approved in certain countries, an anti-dengue virus drug is still needed. Fruits and vegetables may be sources of compounds with medicinal properties, such as flavonoids. This study demonstrates the anti-dengue virus activity of the citrus flavanone naringenin, a class of flavonoid. Naringenin prevented infection with four dengue virus serotypes in Huh7.5 cells. Additionally, experiments employing subgenomic RepDV-1 and RepDV-3 replicon systems confirmed the ability of naringenin to inhibit dengue virus replication. Antiviral activity was observed even when naringenin was used to treat Huh7.5 cells 24 h after dengue virus exposure. Finally, naringenin anti-dengue virus activity was demonstrated in primary human monocytes infected with dengue virus sertoype-4, supporting the potential use of naringenin to control dengue virus replication. In conclusion, naringenin is a suitable candidate molecule for the development of specific dengue virus treatments.

Dengue virus (DENV) infection is a rapidly spreading viral disease worldwide and is transmitted by the *Aedes aegypti* and *Aedes albopictus*^1^ mosquitoes. Two and a half billon people live in dengue-endemic regions in tropical and subtropical countries in Southeast Asia, the Pacific and the Americas^1^. Approximately 390 million dengue cases are estimated each year^2^. In the Americas alone, 2.3 million cases were reported between 2008 and 2012^1^.

Dengue is caused by infection with one of four antigenically related viruses (DENV-1, -2, -3 and -4). The virus, belonging to the genus *Flavivirus* and family Flaviviridae, is a small, enveloped virion with a single 11-kb positive RNA strand. The RNA genome comprises 10 genes, 5′-C-prM-E-NS1-NS2A/B-NS3-NS4A/B-NS5-3′, which are flanked by a 5′ and a 3′ UTR (untranslated region). Infection with DENV leads to dengue fever (DF) without warning signs, DF with warning signs or severe dengue with capillary leakage and hemorrhagic manifestations^1^,^3^,^4^.

Despite the development and approval (in certain countries) of a tetravalent dengue vaccine, antiviral research is still necessary to develop treatments for individuals that are unable to receive the vaccine and for unvaccinated infected patients^5^. The use of plant-derived extracts and their purified compounds as possible antiviral therapies has been explored^6^,^7^. Among various natural products, flavonoids are an important source of compounds possessing antiviral activity against HIV-1, rotavirus, norovirus, adenovirus, herpesvirus (1 and 2), dengue and influenza^8^,^9^,^10^,^11^,^12^,^13^,^14^. The basic structure of a flavonoid consists of a flavan nucleus with 15 carbon atoms arranged in three rings (C-6–C-3–C-6), referred to as A, B, and C. Flavonoid classes include flavones, flavanone, flavonols, flavanolol, isoflavones, flavan-3-ols, anthocyanidins, chalcones and aurones, which differ in terms of their oxidation levels and C ring substitution patterns. Substitutions in the A and B rings differentiate each compound within a class^15^. Flavonoids are widely found in fruits (grapefruit and oranges), vegetables, nuts, seeds, flowers, teas and wine^16^,^17^,^18^,^19^. One flavanone, naringenin, possesses anticancer, antimutagenic, anti-inflammatory, analgesic, antioxidant, antiproliferative and antiatherogenic properties^20^,^21^,^22^. Importantly, naringenin exerts antiviral effects against Hepatitis C virus (HCV), another flavivirus, by inhibiting NS2 activity^23^.

Using flow cytometry, virus titration and replicon assays, this study demonstrated the ability of naringenin to inhibit the replication of four DENV serotypes in Huh7.5 cells. Antiviral activity was evident even when naringenin was used to treat Huh7.5 cells 24 h after DENV infection. To extend our observations to a more relevant system, the antiviral activity of naringenin was also demonstrated in primary human monocytes (CD14^+^) after DENV-4 infection.

## Results

### Naringenin inhibits infection with four different DENV serotypes in Huh7.5 cells

The cytotoxicity of naringenin is cell line-dependent. Different results have been observed in Vero and Hep2 cells^12^,^24^. Therefore, prior to the *in vitro* assessment of antiviral activity in Huh7.5 cells, cytotoxicity was evaluated using the neutral red assay^25^. Plotting cell viability against eight different concentrations of naringenin revealed concentration-dependent toxicity in Huh7.5 cells (Supplementary Figure 1). Also, a naringenin concentration of 311.3 μM inhibited 50% of cell viability (CC_50_). The non-toxic concentration (NTC) of naringenin was 250 μM, which did not differ statistically from untreated control cells.

**Figure 1:**
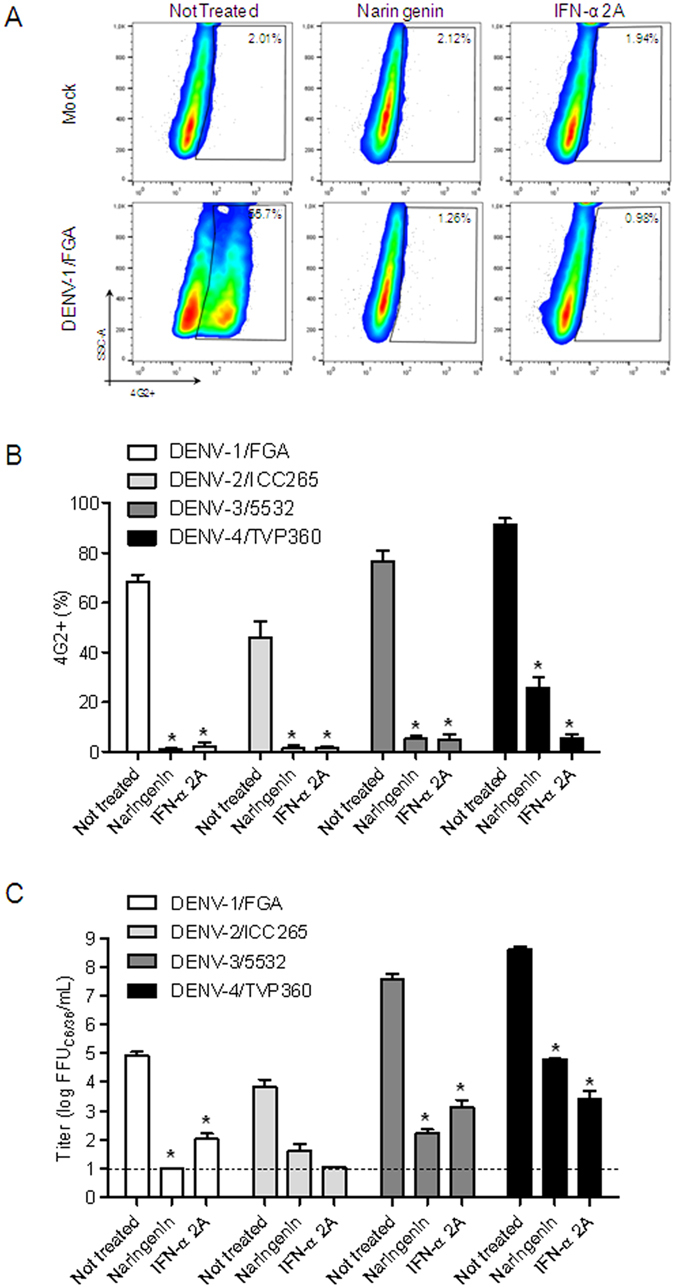
Naringenin inhibits infection by four DENV serotypes in Huh7.5 cells. Representative dot plot (SSC × 4G2) analysis of Huh7.5 cells infected with DENV-1 (FGA/89) serotype after 72 h of treatment with naringenin (250 μM) or IFN-α 2A (200 IU/mL) (**A**). Flow cytometry data from three independent experiments representing the mean ± standard error (SEM) of Huh7.5 cells infected with the four DENV serotypes treated with naringenin or IFN-α 2A (**B**). The cell culture supernatants were titrated in C6/36 cells in a foci-forming immunodetection assay (**C**). Data represent the mean ± SEM from three independent experiments. One-way ANOVA and Dunnett’s test for multiple comparisons (*p < 0.05 compared to DENV control).

**Figure 2:**
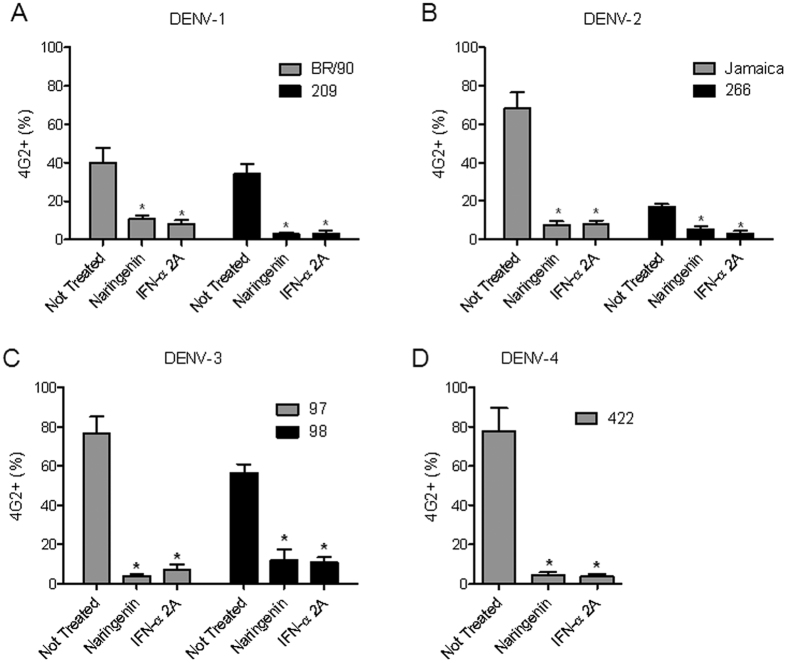
Naringenin inhibits infection by different DENV strains in Huh7.5 cells. Huh7.5 cells were infected with two additional strains each of DENV-1 (**A**), DENV-2 (**B**) and DENV-3 (**C**) and one additional DENV-4 strain (**D**) for 72 h, followed by treatment with naringenin (250 μM) or IFN-α 2A (200 IU/mL). The data represent the mean ± SEM from three independent experiments. One-way ANOVA and Dunnett’s test for multiple comparisons (*p < 0.05 compared to DENV control).

The anti-dengue activity of naringenin was evaluated using four DENV serotypes and recombinant IFN-α 2A as the positive control. Naringenin (250 μM) was added to Huh7.5 cells during and after DENV infection to investigate the entire life cycle of the virus. After 72 h, a flow cytometry assay (FACS) revealed a reduction in the number of DENV-infected cells compared to non-treated controls (Fig. 1A,B). Additionally, the titration of DENV viable particles in cell culture supernatants using a foci-forming immunodetection assay confirmed the anti-DENV activity of naringenin (Fig. 1C). To further explore the anti-DENV effects of naringenin, seven other DENV strains belonging to the four DENV serotypes were tested. As shown in Fig. 2, the percentage of 4G2-positive Huh7.5 cells was reduced after treatment with either naringenin or IFN-α 2A for all strains tested (Fig. 2). Based on these results, the anti-DENV activity of naringenin is independent of the virus serotype or strain.

Furthermore, a concentration-response curve was performed to establish whether the activity in the antiviral assay was dependent on the amount of naringenin. According to our results, the anti-DENV activity of naringenin was concentration-dependent (Supplementary Figure 2 and Table 1). The 50% inhibitory concentration (IC_50_) was calculated, as well as the selectivity index for each DENV serotype (SI = CC_50_/IC_50_).

### Naringenin inhibits DENV replication in Huh7.5 cells

**Figure 3:**
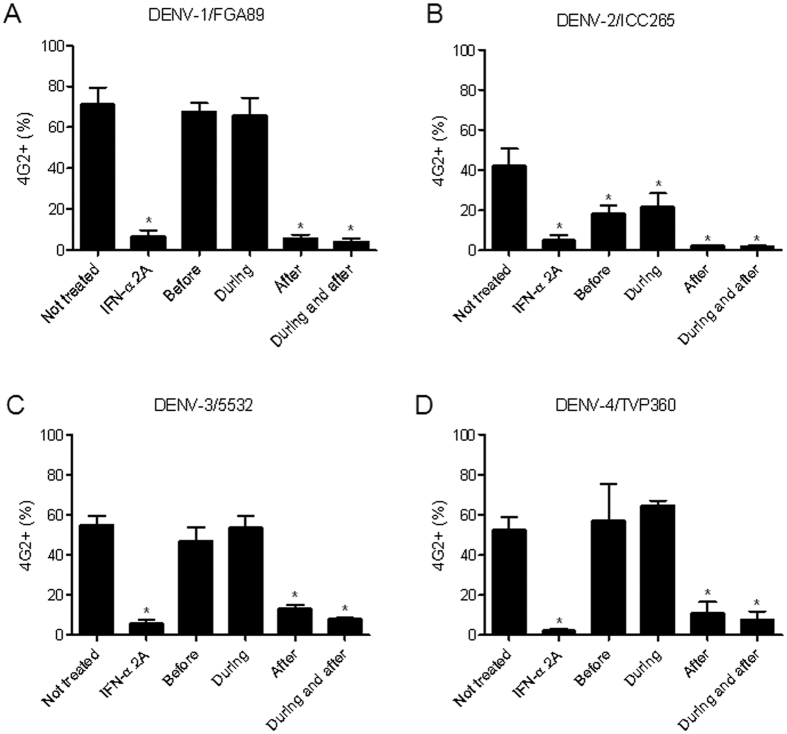
Time-of-drug addition experiments with naringenin. Huh7.5 cells were infected with DENV-1/FGA/89 (**A**), DENV-2/ICC265 (**B**), DENV-3/5532 (**C**) and DENV-4/TVP360 (**D**) and treated with naringenin (250 μM) 1.5 h before infection, during infection, after infection, and during and after infection. As a control, cells were treated with IFN-α 2A (200 IU/mL) after DENV infection. The data represent the mean ± SEM of three independent experiments. One-way ANOVA and Dunnett’s test for multiple comparisons (*p < 0.05 compared to DENV control).

**Figure 4:**
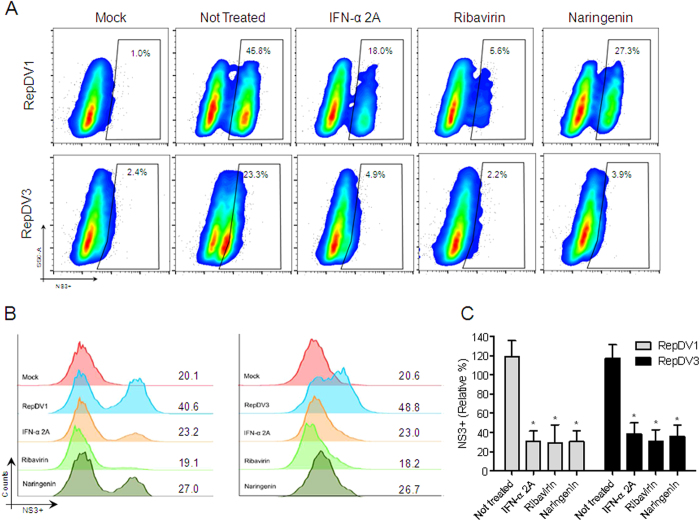
The effects of naringenin on DENV replication. Huh7.5 cells were transfected with either DENV-1 replicon (RepDV1) or DENV-3 replicon (RepDV3) RNA. After 1 h, the transfected cells were treated with naringenin (250 μM). Dot plot analysis (SSC × NS3) of Huh7.5 cells transfected with RepDV1 and RepDV3 treated with naringenin, ribavirin or IFN-α 2A (anti-NS3 staining using the monoclonal antibody 1722) (**A**). Histograms show the mean fluorescent intensity (MFI) of transfected cells treated with naringenin, ribavirin and IFN-α 2A or left untreated (**B**). Average frequency of Huh7.5 cells transfected with RepDV1 or RepDV3 treated with naringenin (**C**). Data represent the mean ± SEM of three independent experiments. One-way ANOVA and Dunnett’s test for multiple comparisons (*p < 0.05 compared to the untreated control).

After demonstrating the antiviral effects of naringenin, we determined the stage of the DENV life cycle during which this compound exerts its effects. Initially, we tested the virucidal effects of naringenin and observed its inability to destroy DENV particles (Supplementary Figure 3). Next, we used the time-of-drug addition approach to observe whether the compound interfered with the early or late phases of the DENV life cycle^26^. In most cases, naringenin treatment before and during infection did not reduce the percentage of Huh7.5 DENV-infected cells, although decreases were observed in the percentages of cells infected with DENV-2/ICC265. Overall, naringenin treatment was most effective either during and after infection or only after infection with the four DENV serotypes. These results were similar to those observed with IFN-α 2A treatment after infection (Fig. 3) and suggest the ability of naringenin to impair DENV replication and/or virus maturation. To determine if naringenin affects viral replication, DENV-1 and -3 replicons (RepDV1 and RepDV3) were employed. These subgenomic RNA systems contain DENV non-structural viral proteins (NS) that enable RNA replication and translation without viable DENV particle assembly. These features make DENV replicons good tools to study virus replication for the purpose of antiviral drug development. The DENV replicon assay is used to identify antiviral compounds that specifically impair DENV replication and/or translation^27^,^28^. According to our results, Huh7.5 cells were successfully transfected, and naringenin reduced DENV replication with an efficiency similar to IFN-α 2A and ribavirin treatments (Fig. 4A–C). Furthermore, DENV-RNA transfection of Huh7.5 cells did not affect cell viability (data not shown).

**Figure 5:**
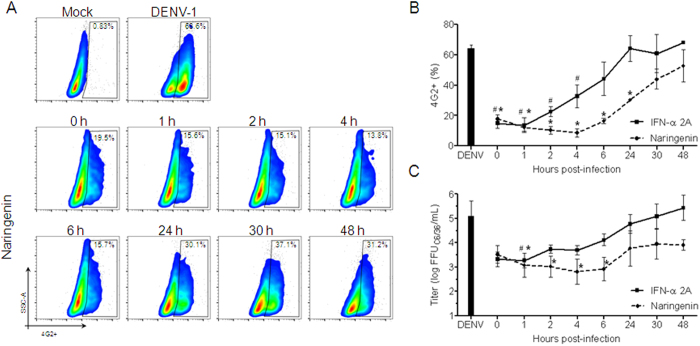
Naringenin treatment after the establishment of DENV infection in Huh7.5 cells. Huh7.5 cells were infected with DENV-1 (FGA/89) and then treated with naringenin (250 μM) or IFN-α 2A (200 IU/mL) at 0, 1, 2, 4, 6, 24, 30 and 48 h post-infection. Dot plot analysis of infected Huh7.5 cells 72 h after infection was established and cells were treated with naringenin at different time points (**A**). Average frequency of Huh7.5 cells infected with DENV-1 and treated with naringenin or IFN-α 2A at different time points after infection (**B**). The cell culture supernatants of infected Huh7.5 cells treated with either naringenin or IFN-α 2A were titrated in C6/36 cells in a foci-forming immunodetection assay (**C**). Data represent the mean ± SEM from three independent experiments. One-way ANOVA and Dunnett’s post-test (*p < 0.05 compared to DENV control).

To determine the efficiency of naringenin after Huh7.5 cells were infected with DENV, treatment was administered at different time points after challenge with the DENV-1 serotype (FGA/89). Naringenin efficiently reduced the percentage of Huh7.5 DENV-1 infected cells even when added 24 h after the infection (Fig. 5). Also, the naringenin treatment 6 h after the DENV-1 infection reduced the DENV-titer at the cell culture supernatant (Fig. 5). Treatment with IFN-α 2A was no longer effective when added 4 h after infection, confirming the potential anti-DENV activity of naringenin (Fig. 5).

### Naringenin decreases DENV infection in human monocytes

**Figure 6:**
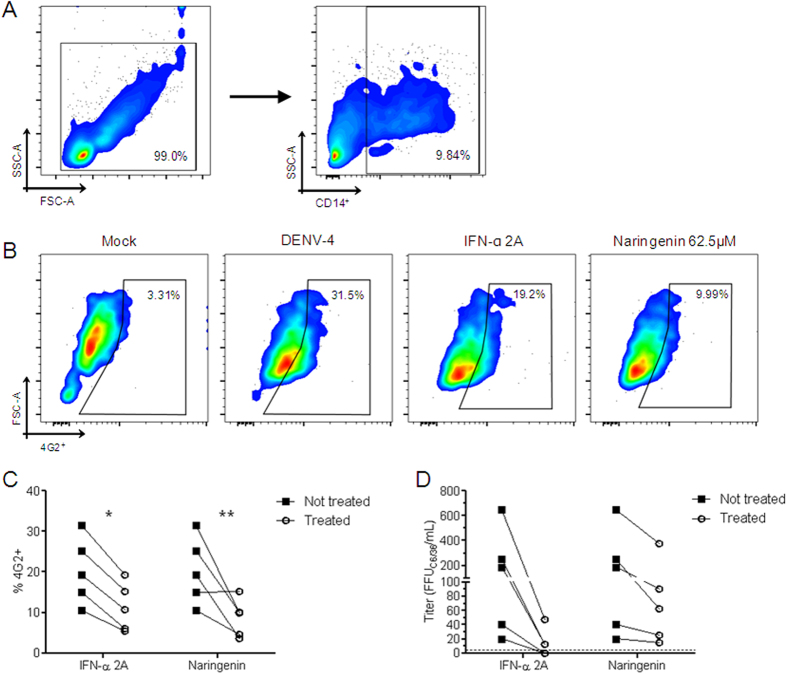
Antiviral activity of naringenin in human monocytes. PBMCs were infected with DENV-4 strain TVP/360 (MOI: 10) and treated with naringenin or IFN-α 2A for five days. Gating strategy showing the selection of viable cells and the percentage of monocytes (CD14^+^) among PBMCs (**A**). Dot plot of DENV-infected monocytes (CD14^+^) showing cell size (FSC) × 4G2 staining of mock and DENV-infected controls and treatment with naringenin (62.5 μM) or IFN-α 2A (200 IU/mL) (**B**). Average frequency of monocytes infected by DENV-4 and treated with naringenin or IFN-α 2A (**C**). The cell culture supernatants of infected monocytes treated with naringenin or IFN-α 2A were titrated in C6/36 cells in a foci-forming immunodetection assay (**D**). Data represent each donor measure and were evaluated by two-way ANOVA and Bonferroni’s post-test (*p < 0.05 compared to DENV control).

Once naringenin anti-DENV activity was confirmed in a human cell line (Huh7.5 cells), the ability of naringenin to impair DENV replication in primary human monocytes from healthy donors was investigated. First, it was established the non-toxic concentration of naringenin for human PBMCs using annexin V/7-AAD assay and monocyte quantification (CD14^+^) (Supplementary Figure 4A–D). Unlike the results in Huh7.5 cells, naringenin was toxic to monocytes at concentrations of 250 and 125 μM but not at 62.5 μM (Supplemetary Figure 4C,D). Thus, as a proof of concept, peripheral blood mononuclear cells (PBMCs) were infected with DENV-4/TVP360 and treated with the non-toxic concentration of naringenin (62.5 μM). Importantly, even at lower concentrations (relative to those employed in Huh7.5 cells), naringenin reduced the number of infected CD14^+^ cells (monocytes) in human PBMCs as well as the number of infectious viral particles in the culture supernatant (Fig. 6A–D).

## Discussion

Flavonoids constitute a class of naturally occurring compounds that possess multiple pharmacological characteristics such as antioxidant, anti-inflammatory, analgesic, anti-carcinogenic, antibacterial infection, antifungal and antiviral properties^20^. The antiviral activity of flavonoids or flavonoid-derived compounds has been demonstrated against several viruses affecting humans, including norovirus, rotavirus, adenovirus, herpes simplex, HCV, dengue, chikungunya and HIV-1^9^,^10^,^11^,^12^,^13^,^14^,^23^,^29^,^30^. Here, we evaluated the anti-DENV activity of the flavanone naringenin.

Flavonoids are amply present in fruits and vegetables. Importantly, flavonoids present low toxicity in different cell lines^24^,^31^. Here, the CC_50_ of naringenin was determined to be 311.3 μM (84.75 μg/mL) in Huh7.5 cells, similar to that reported by Zandi *et al*.^12^ in Vero cells (CC_50_ of 304.85 μM; 83 μg/mL)^12^. We also established the NTC of naringenin in Huh7.5 cells (250 μM; 68.06 μg/mL). In a previous study by Khachatoorian *et al*.^32^, naringenin was not toxic to Huh7.5 cells at concentrations between 25 and 125 μM^32^. However, for primary human monocytes, a lower concentration of naringenin had to be used (62.5 μM; 16.94 μg/mL). Additionally, Zanello *et al*.^33^ studying anti-dengue virus activity of quinic acid derivatives, had demonstrated that primary human PBMC seems to be more sensible than cell lines, confirming our observation^33^.

The flavonoid fisetin inhibits DENV-2 infection in Vero cells, whereas naringenin and rutin do not^12^. Additionally, naringenin has virucidal effects on DENV-2^12^. Surprisingly, using flow cytometry and virus titration, we demonstrated the ability of naringenin to efficiently inhibit infection by eleven different DENV strains representing the four different virus serotypes in the Huh7.5 cell line. In contrast to results reported by Zandi *et al*.^12^, our RT-PCR assay results did not reveal any virucidal effects of naringenin on any of the four DENV serotypes^26^,^34^. Zandi *et al*.^12^ only used the DENV-2 strain (New Guinea C), while we employed an RT-PCR assay and recent clinical isolates of the four different DENV serotypes to investigate the virucidal effects of naringenin. These differences may explain the contradictory results.

To determine the stage of the DENV life cycle during which naringenin exerts its antiviral effects, time-of-drug addition experiments were carried out^26^. Aside from the partial inhibition observed with DENV-2, naringenin did not affect binding and entry into Huh7.5 cells. Indeed, naringenin appeared to target other stages of the DENV life cycle, including replication and/or maturation. To determine if naringenin impairs DENV replication, we used the DENV-1 and DENV-3 replicon systems^27^,^28^. In accordance to quinic acid derivatives and 2-bromo-α-ergocriptine, naringenin appears to reduce DENV replication^33^,^35^. Recently, a stable BHK-21 cell line carrying the chikungunya replicon was employed to demonstrate the ability of naringenin to impair chikungunya virus (CHIKV) replication^31^. It seems that some flavonoids could impair the activity of viral proteins important for virus replication, like the serine protease activity (NS2B-NS3) of dengue and Zika virus^36^,^37^, and also the protease (NS2) of HCV^23^. Furthermore, naringenin inhibits intracellular HCV protein production and viral assembly, in agreement with our findings^32^. Flavonoids also impair influenza A replication and spread^38^. C2 group replacement in the chemical bond between the C2 and C3 rings may interfere with the cellular response to influenza A infection via MAPK signaling pathway modulation^38^.

Even when naringenin was added to Huh7.5 cell cultures several hours after infection (6 h and 24 h), the synthesis of viable virus particles and the percentage of infected cells were significantly reduced. Naringenin controlled DENV replication as efficiently as IFN-α 2A, a known antiviral cytokine^39^. 2-bromo-α-ergocriptine impairs DENV translation and/or RNA synthesis as late as 6 h after infection^35^. Treatment of DENV-2-infected cells with NITD-618, a benzomorphan core structure, reduces infection even when administered 12 h after infection^40^,^41^. The effects of naringenin 24 h after DENV infection in Huh7.5 cells demonstrated the potential of this flavanone as an anti-DENV compound.

In addition to the Huh7.5 cell line, the antiviral effects of naringenin were also tested in PBMCs isolated from healthy individuals and infected *in vitro*. Naringenin efficiently reduced infection in primary human monocytes, the primary cell targets of DENV^42^. Thus, the antiviral effects of naringenin were also observed in primary human monocytes, reinforcing the data obtained in the cell line. However, more studies are needed in order to better understand the mechanism of naringenin action at these cells.

In conclusion, data from multiple assays (flow cytometry, viral titration and replicon system) employed to assess infection with eleven different strains representing the four DENV serotypes in two cell types (the Huh7.5 cell line and primary human monocytes) support the ability of naringenin to target DENV replication, making naringenin a suitable candidate for the treatment of DENV infection. Our results provide novel insights for the development of specific anti-DENV drugs to treat infected patients.

## Materials and Methods

### Cells, DENV and Naringenin

*Aedes albopictus* mosquito cells C6/36 (ATCC: CLR-1660) were grown in Leibovitz’s Medium (L-15; Gibco, Grand Island, NY, USA) supplemented with 0.26% tryptose (Sigma-Aldrich, St. Louis, MO, USA), 5% fetal bovine serum (FBS; Gibco, Grand Island, NY, USA) and 25 μg/mL gentamicin (Sigma-Aldrich, St. Louis, MO, USA) at 28 °C. The human-derived Huh7.5 hepatoma cell line (ATCC PTA-8561) was grown in Dulbecco’s Modified Eagle Medium/nutrient mixture F-12 (D-MEM/F-12; Gibco, Grand Island, NY, USA) supplemented with 100 IU/μg/mL penicillin/streptomycin (Gibco, Grand Island, NY, USA) and 10% FBS at 37 °C in a humidified, 5% CO_2_ controlled atmosphere.

PBMCs were isolated from the whole blood of healthy volunteers (approved by the FIOCRUZ Committee of Ethics in Research, number 514/09), by density gradient centrifugation in lymphocyte separation medium (Lonza, Walkersville, MD, USA). PBMCs were cultured in 24-well plates with Roswell Park Memorial Institute medium-1640 (RPMI-1640; Lonza, Walkersville, MD, USA) supplemented with 2 mM glutamine (Gibco, Grand Island, NY, USA), 100 IU/μg/mL penicillin/streptomycin, 2.5 μg/mL amphotericin B (Sigma-Aldrich, St. Louis, MO, USA), 100 mM sodium pyruvate (Sigma-Aldrich, St. Louis, MO, USA) and 10% FBS in a humid, 37 °C atmosphere with 5% CO_2_. The authors confirm that all experiments were performed in accordance with relevant guidelines and regulations.

DENV-1/FGA/89 was isolated in 1989 from a South American patient suffering from DF (GenBank: AF226687). DENV-2/ICC-265 was isolated from a DF patient in Brazil in 2009. DENV3/5532 was isolated in 2007 from a fatal case of dengue with visceral manifestations in a patient in Paraguay (GenBank: HG235027). DENV-4/TVP360 is a laboratory strain that was kindly provided by Dr. Ricardo Galler (Fundação Oswaldo Cruz, Rio de Janeiro, Brazil; GenBank: KU513442). Other DENV virus strains from Brazilian patients with DF between 1990 and 2014 were also used: DENV-1/BR/90 (GenBank AF226685.2), DENV-1 209, DENV-2 Jamaica, DENV-2/ICC-266, DENV-3/97 (GenBank EF629367), and DENV-3/98 (GenBank EF629368). Additionally, a clinical isolate from a non-fatal case of DENV-4 with hemorrhagic manifestations was employed (DENV-4/LRV13/422; GenBank: KU513441)^43^. Virus stocks were propagated in C6/36 cells and titrated in a foci-forming immunodetection assay^44^.

Naringenin (≥95% purity) was purchased from Santa Cruz Biotechnology and prepared in a 100% solution of dimethyl sulfoxide (DMSO, Sigma-Aldrich, St. Louis, MO, USA).

### Cytotoxicity Assays

Huh7.5 cell viability was tested using a neutral red (NR; Sigma-Aldrich, Irvine, UK) uptake assay as previously described^25^. Serial dilutions of naringenin (50 mM stock) in DMEM-F12 medium and 1% DMSO were added to 2 × 10^4^ Huh7.5 cells/well in a 96-well plate and incubated for 72 h. The supernatant was removed, and a solution containing neutral red (33 μg/mL) was applied for 2 h. After incubation, the supernatant was removed, and an acidified ethanol solution was used to extract the dye and perform optical density (OD) quantification at an absorbance of 540 nm using a spectrophotometer. Data from three independent experiments were normalized using the following equation: cell viability (%) = (OD sample value − OD blank control)/(OD cell control − OD blank control) × 100. The NTC was defined as the highest concentration that did not differ statistically from untreated control cells. The concentration that inhibited viability in 50% of cells (CC_50_) was obtained by performing nonlinear regression followed by the construction of a sigmoidal concentration-response curve (variable slope; GraphPad Prism; La Jolla, CA, USA).

### Antiviral Activity Assays

The antiviral activity of naringenin was assessed in two different assays. Huh7.5 cells at concentration of 2 × 10^4^ cells/well in 96-well plates were infected with one representative strain of each of the four DENV serotypes (DENV-1/FGA/89, DENV-2/ICC265, DENV-3/5532 and DENV-4/TVP360) at a multiplicity of infection (MOI) of 10 for 90 minutes. Naringenin at its NTC was added during and after infection. Positive and negative controls for antiviral activity were included using IFN-α 2A (200 IU/mL; Blau Farmacêutica, Cotia, SP, Brazil) and mock-infected cells, respectively. After 72 h of incubation, the cell culture supernatants were stored at −86 °C for virus titration. The cells were detached and stained for flow cytometry^33^. A BD FACS Canto II (BD Biosciences, USA) was used to quantify cells infected by DENV. Cell culture supernatants were also employed for titration in a foci-forming immunodetection assay in C6/36 cells to confirm the FACS results^44^.

Additionally, dose response curves were obtained with a serial dilution starting at the NTC of naringenin. The concentration that inhibited 50% of virus infection (IC_50_) was obtained using nonlinear regression, followed by the construction of a sigmoidal concentration-response curve (variable slope; GraphPad) and calculation of the selectivity index (SI = CC_50_/IC_50_). All assays were performed in triplicate.

Antiviral activity was further confirmed for other DENV strains representing the four serotypes (DENV-1 209, DENV-1/BR/90, DENV-2/ICC-266, DENV-2 Jamaica, DENV-3/97, DENV-3/98 and DENV-4/LRV13/422) using the FACS assay as described above.

### Virucidal Assay

A virucidal assay was performed as previously described with minor modifications^34^. Briefly, a sample of each DENV serotype (2 × 10^5 ^ffu/mL; DENV-1/FGA/89; DENV-2/ICC-265; DENV3/5532 and DENV-4/TVP360) was treated with naringenin (250 μM) in the presence or absence of 150 μg/mL RNase A (USB-Affymetrix; Santa Clara, CA, USA) for 1 h at 37 °C. After treatment, viral RNA was extracted using a QIAamp Viral RNA Mini Kit (QIAGEN; Hilden, Germany). The RNA was reverse transcribed using 250 pmol of a random primer (Gibco, Grand Island, NY, USA) and Improm II Reverse Transcriptase (Promega, Fitchburg, WI, USA). PCR amplification was performed as described by Lanciotti *et al*.^45^ with a D1 (5′-TCAATATGCTGAAACGCGCGAGAAACCG-3′) and D2 (5′-ATTGCACCAGCAGTCAACGTCATCTGGTTC-3′) primer pair. DENV RNA samples treated with RNase or left untreated were used as the positive and negative control, respectively.

### Time-of-Drug Addition Assay

Initially, Huh7.5 cells at a density of 2 × 10^4 ^cells/well in 96-well plates were (i) treated with naringenin for 1 h prior to DENV infection; (ii) treated with naringenin during DENV infection; (iii) treated with naringenin after DENV infection; or (iv) treated with naringenin during and after DENV infection. An MOI of 10 was used for DENV infection, and naringenin was tested at a concentration of 250 μM. The percentage of infected cells was determined using FACS as previously described.

In another experiment, cells were infected with DENV-1 strain FGA/89 (MOI: 10) as previously described and treated with naringenin (250 μM) or with IFN-α 2A (200 IU/mL) at different time points after infection (0, 1, 2, 4, 6, 24, 30 and 48 h). The percentage of infected cells was determined using FACS. The supernatants were titrated in a foci-forming immunodetection assay 72 h post-infection.

### Naringenin Impairs DENV Replication in Huh7.5 Cells

After confirming the anti-DENV effects of naringenin after viral entry into Huh7.5 cells, we determined if this flavanone impairs DENV replication. Two subgenomic replicons derived from DV1-BR/90 (RepDV1) and BR DEN3 290-02 (RepDV3) were used in these experiments^27^,^28^. DENV replicon RNAs were obtained and used to transfect Huh7.5 cells (2 μg of RNA/2 × 10^6^ cells) in a Nucleofector 2B device (Lonza; Cologne, Germany) according to the manufacturer’s instructions^33^. After 1 h of transfection, the Huh7.5 cells were treated with naringenin (250 μM). The plates were further incubated for 72 h. After incubation, the cells were recovered and subjected to FACS analysis, as described previously. As a negative control, Huh7.5 cells were transfected without RNA and were not treated. Huh7.5 cells transfected with RNA (from the DENV-1 or DENV-3 replicon) and untreated cells were used as positive controls for virus replication. The reference control consisted of treatment with recombinant IFN-α 2A (200 IU/mL) and 20 μM ribavirin (Sigma-Aldrich, St. Louis, MO, USA).

### Antiviral Effects in Primary Human Cells

PBMCs obtained from healthy donors who provided signed informed consents were purified using Ficoll-histopaque and a classical protocol^46^. After purification, PBMCs were plated onto 24-well plates at a density of 1 × 10^6 ^cells/well and treated with 200 μM of naringenin for 5 days and then the exposure of annexin V/7-AAD was evaluated. Also, we quantified the number of human monocytes (CD14^+^) after treatment with naringenin (250, 125 and 62.5 μM) for five days. After it was established that the NTC of naringenin for human monocytes was 62.5 μM, infection with dengue was performed. Human PBMCs were plated onto 24-well plates at a density of 1 × 10^6 ^cells/well and infected with DENV-4 TVP/360 (MOI: 10) for 2 h. The inoculum was removed, and cells were treated with naringenin (62.5 μM), IFN-α 2A (200 IU/mL) or RPMI media. After incubation for 5 days at 37 °C and 5% CO_2_, cells were stained for DENV E protein (mAb 4G2-FITC conjugated) and with a mouse anti-human CD14 PE-Cy7 antibody^47^ (BD Biosciences, USA). CD14 expression was used to gate the monocytes. The percentage of DENV E-positive cells (4G2^+^) in this population was calculated.

### Data Analysis

Statistical analysis consisted of one-way ANOVA followed by Dunnett’s test for multiple comparisons with a significance of p < 0.05. Statistical analyses were performed using Prism software (GraphPad version 5.0; La Jolla, CA, USA). Primary human monocyte infection was analyzed using two-way ANOVA and the Bonferroni correction for multiple comparisons with a significance level of p < 0.05. Flow cytometry data were analyzed using FlowJo version X software (Tree Star Inc.; Ashland, OR, USA).

## Additional Information

**How to cite this article:** Frabasile, S. *et al*. The citrus flavanone naringenin impairs dengue virus replication in human cells. *Sci. Rep.* **7**, 41864; doi: 10.1038/srep41864 (2017).

**Publisher's note:** Springer Nature remains neutral with regard to jurisdictional claims in published maps and institutional affiliations.

## Supplementary Material

Supplementary Information

## Figures and Tables

**Table 1:** Naringenin half maximal inhibitory concentration (IC_50_) and selectivity index (SI) for anti-DENV activity in Huh7.5 cells.

DENV	IC_50_	SI
DENV-1/FGA	35.81	8.60
DENV-2/ICC265	17.97	17.32
DENV-3/5532	117.1	2.66
DENV-4/TVP360	177.5	1.75

SI = CC50/IC50.
