# Corrigendum: The citrus flavanone naringenin impairs dengue virus replication in human cells

**DOI:** 10.1038/srep43976

**Published:** 2017-03-14

**Authors:** Sandra Frabasile, Andrea Cristine Koishi, Diogo Kuczera, Guilherme Ferreira Silveira, Waldiceu Aparecido Verri, Claudia Nunes Duarte dos Santos, Juliano Bordignon

Scientific Reports 7: Article number: 4186410.1038/srep41864; published online: 02 03 2017; updated: 03 14 2017

In the original version of this Article, Claudia Nunes Duarte dos Santos and Juliano Bordignon were incorrectly affiliated with ‘Sección Virologia, Facultad de Ciencias, Universidad de La República, 11400, Montevideo, Uruguay’. The correct affiliations are listed below:

Claudia Nunes Duarte dos Santos

Laboratório de Virologia Molecular, Instituto Carlos Chagas, ICC/FIOCRUZ/PR, Curitiba, Paraná, Brazil

Juliano Bordignon

Laboratório de Virologia Molecular, Instituto Carlos Chagas, ICC/FIOCRUZ/PR, Curitiba, Paraná, Brazil

These errors have now been corrected in the PDF and HTML versions of the Article.

