# Supplementary material for: The citrus flavanone naringenin impairs dengue virus replication in human cells
Source: Sci Rep. 2017 Feb 3;7:41864. doi: 10.1038/srep41864 (PMC5291091; doi:10.1038/srep41864)
Supplement: Supplementary Information [file srep41864-s1.pdf]

Supplementary Information:

The citrus flavonone naringenin impairs dengue virus replication in human cells

Sandra Frabasile[1,2#], Andrea Cristine Koishi[2#], Diogo Kuczera[2], Guilherme Ferreira Silveira[2], Waldiceu Aparecido Verri, Jr[3], Claudia Nunes Duarte dos Santos[1], Juliano Bordignon[1*]

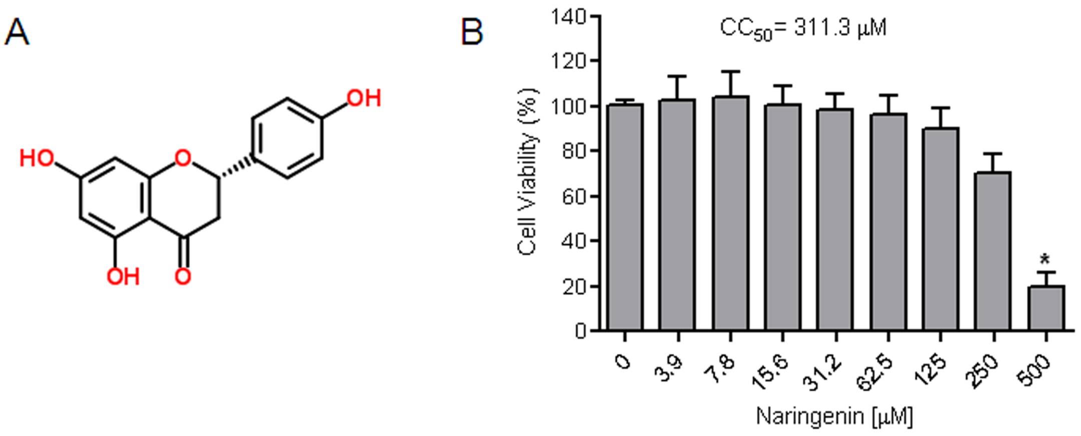

S. Figure 1: Chemical structure and cytotoxicity of naringenin. Chemical structure of the flavonone naringenin (A). Different concentrations of naringenin were used to treat Huh7.5 cells to determine cytotoxicity using a neutral red assay (B). Data represent the mean ± SEM from three independent experiments. One-way ANOVA and Dunnett's test for multiple comparisons (*p<0.05 compared to not treated control) (B). The concentration that inhibited 50% of cell viability (CC_{50}) was obtained by performing nonlinear regression followed by the construction of a sigmoidal concentration-response curve (variable slope; GraphPad).

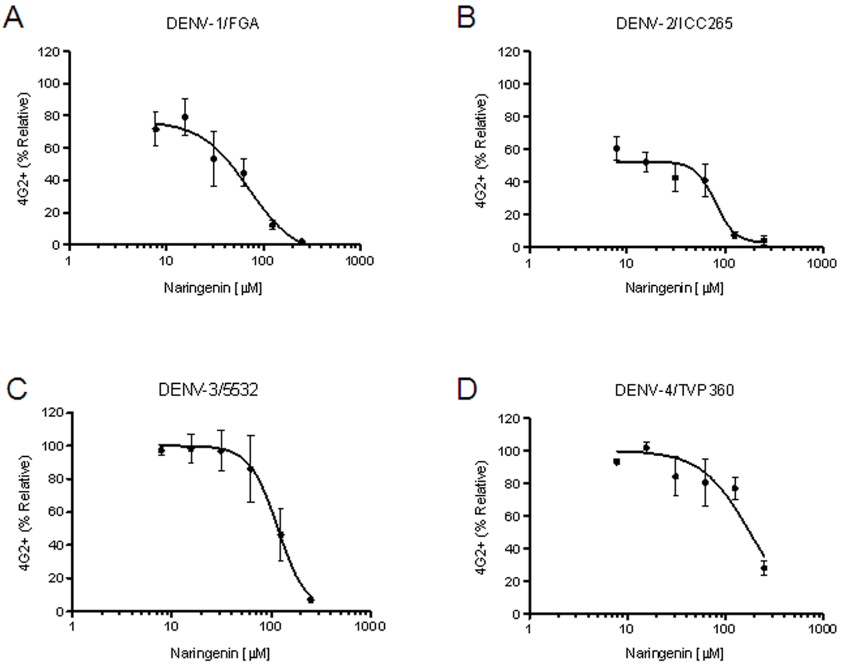

S. Figure 2: Concentration response curve of naringenin against the four DENV serotypes. Huh7.5 cells were infected with DENV-1/FGA89 (A), DENV-2/ICC265 (B), DENV-3/5532 (C) and DENV-4/TVP360 (D) and treated after infection with a range of naringenin concentrations (250 – 7.8 μM). Naringenin concentrations that inhibited infection in 50% of Huh7.5 cells were defined using a sigmoidal dose response curve (variable slope). Data represent the mean ± SEM of three independent experiments.

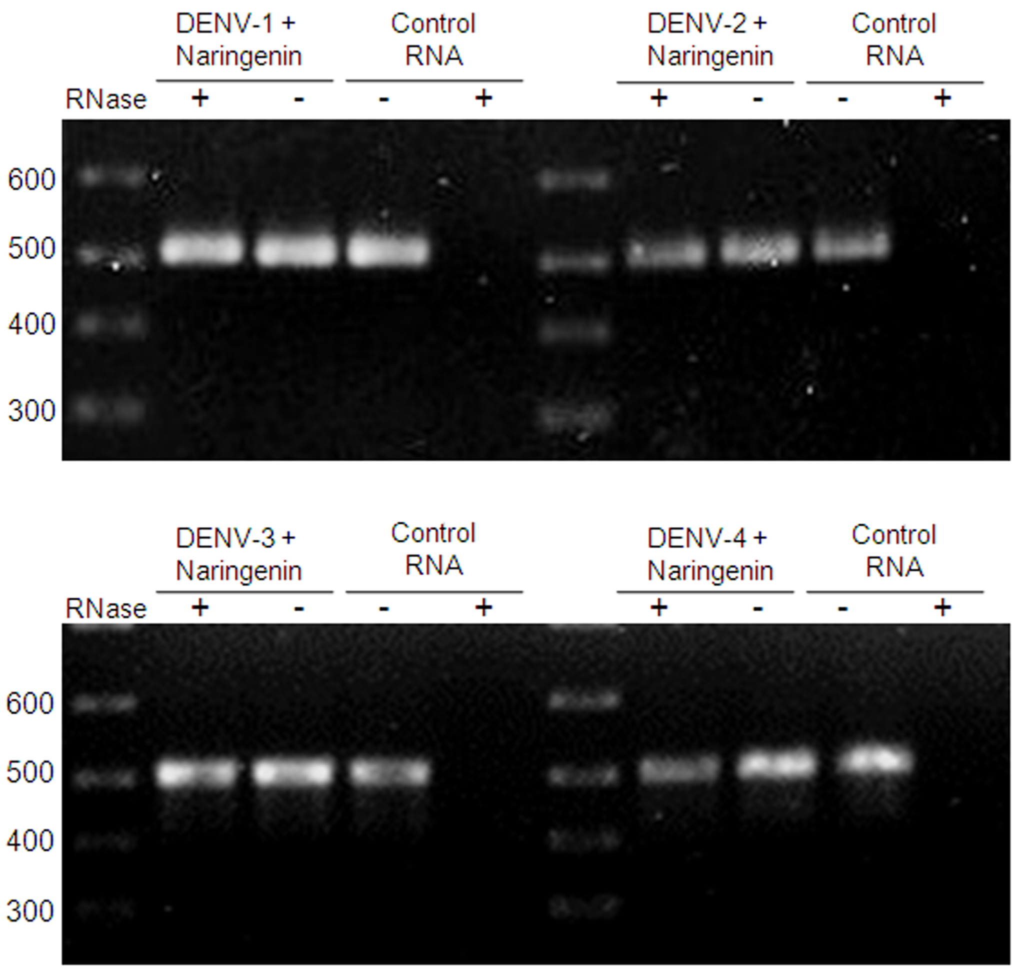

S. Figure 3: Evaluation of naringenin virucidal activity. The four DENV serotypes (DENV-1/FGA/89; DENV-2/ICC-265; DENV3/5532 and DENV-4/TVP360) were incubated with naringenin (250 μM) in the presence or absence of RNase. RNA samples were extracted and subjected to RT-PCR and gel electrophoresis. Representative data from 4 independent assays are shown. M: 1 kb DNA ladder; RNA; viral RNA control; bp: base pairs.

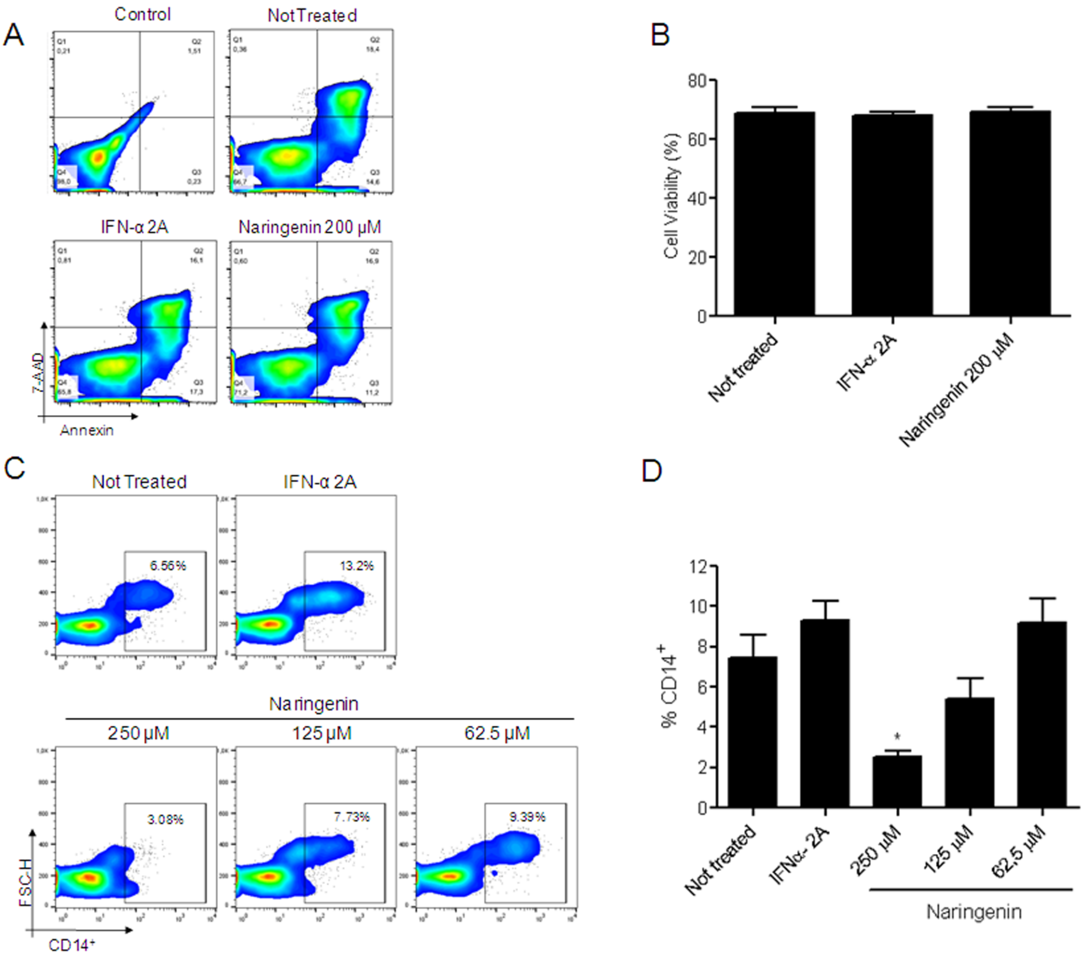

S. Figure 4: Cytotoxicity of naringenin in primary human monocytes. PBMCs were treated with 200 μM naringenin or IFN-α 2A, and the expression of annexin V was determined after 72 h (A). Average number of annexin V monocytes from six healthy donors after treatment with naringenin or IFN-α 2A (B). Representative dot plot of one healthy donor showing the number of $CD14^+$ monocytes after treatment with naringenin (250 – 62.5 μM) (C). Average number of $CD14^+$ cells after treatment with different concentrations of naringenin or 200 IU/mL IFN-α 2A (D). Data represent the mean ± SEM from three donors. One-way ANOVA and Dunnett's test for multiple comparisons (*$p<0.05$ compared to DENV control).
